# Design of Waterborne Asymmetric Block Copolymers as Thermoresponsive Materials

**DOI:** 10.3390/polym12061253

**Published:** 2020-05-30

**Authors:** Gordana Siljanovska Petreska, Christof van Sluijs, Clemens Auschra, Maria Paulis

**Affiliations:** 1 POLYMAT, University of the Basque Country UPV/EHU, Joxe Mari Korta Center, Avda. Tolosa 72, 20018 Donostia-San Sebastián, Spain; gordana.siljanovska-petreska@basf.com; 2 BASF SE, 67056 Ludwigshafen, Germany; clemens.auschra@basf.com; 3 BASF Nederland BV, 8440 AJ Heerenveen, The Netherlands; christof.van-sluijs@basf.com

**Keywords:** RAFT, miniemulsion polymerization, AB hard-soft block copolymers, particle and film morphology, heat seal lacquers

## Abstract

AB diblock waterborne copolymers made of styrene (St) and 2-ethylhexyl acrylate (2EHA) were synthesized by means of two-step reversible addition fragmentation chain transfer (RAFT) (mini)emulsion polymerization. Monofunctional asymmetric RAFT agent was used to initiate the polymerization. The hard polystyrene “A” block was synthesized via miniemulsion polymerization followed by 2EHA pre-emulsion feeding to form the soft “B” block. Polymerization kinetics and the evolution of the molecular weight distribution were followed during synthesis of both initial and final block copolymers. DSC measurements of the block copolymers revealed the existence of two glass transition temperatures (Tgs) and thus the occurrence of two-phase systems. Microscopic techniques (atomic force microscopy (AFM) and transmission electron microscopy (TEM)) were used to study the phase separation within the particles in the latex form, after film formation at room temperature cast directly from the latex and after different post-treatments well above the Tg of the hard-polystyrene domains, when complete particle coalescence had occurred. The morphological differences observed after different annealing temperatures were correlated with the mechanical properties analyzed by DMTA measurements. Finally, the differences found in the mechanical properties of the block copolymers annealed at different temperatures were correlated to their heat seal application results.

## 1. Introduction

**Figure 1:**
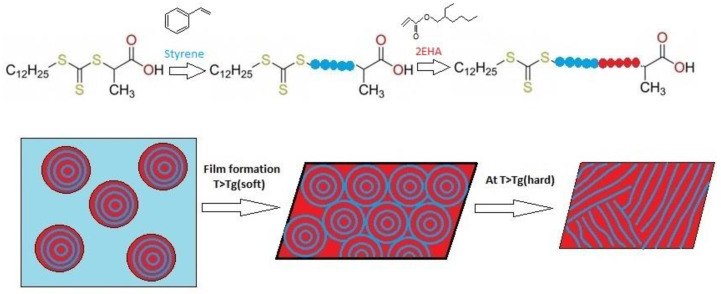
Chemical structure of the BM1430 reversible addition fragmentation transfer (RAFT) agent, and the expected effect of the thermoresponsiveness on the morphology of the microphase separated hard-soft block copolymers.

The synthesis of block copolymers bearing polymers with different properties (i.e., different glass transition temperatures (Tgs)), provides a great opportunity to obtain chemical linkages between polymer chains that could otherwise be completely phase separated. Three main types of reversible-deactivation radical polymerizations (RDRPs)—(i) nitroxide mediated polymerization (NMP) [1], (ii) atom transfer radical polymerization (ATRP) [2], and (iii) reversible addition fragmentation transfer (RAFT) polymerization [3,4]—were developed and received great attention for the synthesis of well-defined block copolymers. Among all the RDRPs, RAFT is the most versatile and robust technique which allows the polymerization of a broader range of functional monomers (both polar and nonpolar) [5]. It has also a higher tolerance to diverse functional groups than do competing techniques (NMP and ATRP), in a wide range of reaction conditions, including bulk, solution, and aqueous dispersions [6,7] using different classes of chain transfer agents (CTAs). The key feature of the RAFT mechanism is a sequence of addition-fragmentation equilibria [8]. When the polymerization is complete (or stopped) most of the chains retain the thiocarbonylthio end group upon completion of the polymerization and can be isolated as stable materials, which can then be extended with the second monomer to build the block copolymer. The most important feature of RAFT polymerization is the structure and selection of the RAFT agent (ZS(=S)SR) [9], which should be done according to the monomers being polymerized and the reaction conditions [10,11,12]. In this study, a 2-(((dodecylthio)carbonothioyl)thio)propanoic acid (BM1430, see Figure 1) RAFT agent was chosen to produce block copolymers of styrene and 2-ethyl hexyl acrylate block copolymers.

RAFT polymerization in bulk and solution has been very successful. However, among all the polymerizations media, aqueous dispersed systems are favored because of low environmental impact, good heat transfer, and low viscosity. When RAFT was initially employed in aqueous dispersed media, problems like (i) poor colloidal stability [13,14], (ii) poor control of the number- average molecular weight [13,14] and (iii) poor control of the dispersity [14] were reported. These problems mainly originated from the difficulties related to the insufficient solubility characteristics of the RAFT agent in water, which has to migrate through the water phase to the locus of polymerization, namely from monomer droplets to polymer particles. Miniemulsion polymerization [15] is a heterogeneous aqueous polymerization process that avoids the transport through the water phase and permits the incorporation of water insoluble compounds. RAFT miniemulsion process has been successfully used for the synthesis of block copolymers of AB [16,17,18] and ABA [17] type block copolymers and the nanostructured particle [19,20,21] morphology produced has been profoundly investigated. However, the direct film formation from the nanostructured aqueous latexes so far has not been extensively investigated. In most studies, polymer films were prepared by casting from a homogenous solution in an organic solvent (i.e., via dissolution of the dried or coagulated latex in a good solvent) [22,23]. In such a solvent casting process, the original particle morphology is removed and can have no impact on the film formation and the resulting morphology of the block copolymer film. Yang et al. [21] were the first authors to synthesize ABA triblock copolymers by RAFT miniemulsion polymerization starting from a macroRAFT and study the morphology of the films obtained directly from the latex. The cast latex films exhibited nanostructured morphologies, which did not attain thermodynamic equilibrium even after thermal annealing. This was attributed to the kinetic limitations on interparticle self-assembly of copolymers. We previously synthesized symmetric ABA block copolymers containing crystalline-soft-crystalline domains [24] and hard-soft-hard domains [25]. The relation between the particle morphology and film formation was studied at different conditions and it was shown that both types of block copolymers attained the thermodynamic equilibrium after thermal annealing of the films cast directly from the latexes. In addition, it was proven that one could tune the mechanical properties of the annealed films by choosing the right monomer, hard or crystalline, and by the amount of the hard phase monomer. However, so far, we have yet to present examples of potential application fields for such thermoresponsive block copolymers.

Block copolymers synthesized via RAFT (mini)emulsion polymerization or other techniques of controlled radical polymerization are mostly described for specialty polymer applications like dispersant or emulsifier additives and not for industrial big volume polymer applications. Orica Consumer Products, for example, produced amphiphilic reactive block copolymers as surfactants to produce latexes with controlled particle size [26]. Furthermore, they extended their invention [27] to the possibility to produce stable latexes and encapsulate pigments like TiO_2_. These applications take advantage of the different chemical nature of the different blocks present in the block copolymers to favor the compatibility of two chemically incompatible materials. However, thermoresponsive block copolymers take advantage of the temperature controlled change in properties of both microphases of the different blocks. The change in properties can be a change in the water solubility of one of the blocks, which leads to upper or lower critical solution temperatures and is usually aimed for biomedical applications [28,29]; however, the temperature can also change the mobility of the polymer chains, as it happens in the glass transition (Tg) or melting temperature of amorphous or crystalline polymer chains. This last type of thermal transition, and the associated thermoresponsiveness [30], are explored in this work. In this article, we present our investigations on the potential use of the microphase separated block copolymers in heat seal lacquers by applying the aqueous latex directly to cast sealing layers onto different substrates. Heat seal lacquers stand for an expedient and effective method to seal paper, foil, and other films for a diversity of packaging materials. In heat seal lacquers a flexible substrate is coated on one side, stored and transported or otherwise processed. The coated substrate is later sealed to another surface with heat and pressure to achieve an effective bonding at the interface. Heat seal lacquers are generally stored in a wound roll or they are stacked in sheets. Thus, the coatings should be carefully designed to find a good compromise between conflicting properties: they require a high Tg to prevent the coating from sticking to the layer above, which could occur during storage at ambient temperature, and at the same time, it is desirable to maintain a low sealing initiation temperature. Water-based dispersions can be specifically designed for many different end-uses due to the ability to create multi-phase particles, amongst which heat seal lacquers are a well-known example. Since different performance criteria are needed at different heat seal steps (first non-adhesive and adhesive upon heating), low and high Tg polymers are desirable in heat-activatable sealants. Many patents and articles [31,32,33,34,35,36,37,38,39] have been published in which authors design dispersions with a core-shell morphology with a higher Tg shell and a lower Tg core. When exposed to heat and pressure above the Tg of the hard phase, diffusion kinetics and chain mobility allow for the mixing and re-arrangement of the phases and for the formation of a continuous softer phase [40]. The substrate surfaces wetted as chains will be mobile and good bond strength will be formed upon cooling. The final bond strength is related to the adhesion of the sealant to each surface as well as the cohesive strength of the sealant layer itself. In terms of production speed, a relatively low seal initiation temperature and plateau initiation temperature of a coating allow for more packages to be sealed in a given time. However, a low seal temperature coating can also be predisposed to blocking in a roll or stack, where the pressure is high enough to cause the coating to stick to the layer above upon storage. This then leads to difficulty in unwinding, surface defects, or equipment jamming during the converting process.

Therefore, in this work we study the heat sealing properties of block copolymers synthesized by RAFT (mini)emulsion polymerization. We synthesize waterborne asymmetric AB block copolymers, where A is the hard domain and B is the soft domain and study the particle morphology and film formation properties of the films annealed at different conditions. In addition, we test the application properties of the block copolymers cast directly from the latex films. The synthesis of such dispersions gives us the freedom to design the balance of the Tg in both phases in such a way as to determine a low seal initiation temperature and at the same time enable good blocking resistance at room temperature.

## 2. Materials and Methods 

### 2.1. Materials

Technical grade monomers styrene (St, 99%–100% purity, inhibitor 4-tert-butylcatechol (TBC)), and 2-ethylhexyl acrylate (2EHA) were used to synthesize the block copolymers. Stearyl acrylate monomer (SA, Sigma Aldrich, Madrid, Spain) was used as a costabilizer to prevent Ostwald ripening. Sodium bicarbonate (NaHCO_3_, Aldrich) was added as a buffer to control the miniemulsion viscosity by reducing the electrostatic interactions among droplets. Alkyldiphenyloxide disulfonate (Dowfax 2A, 45 wt.% active content, DOW Chemicals, Midland, MI, USA) and Disponil A3065 (65 wt.% active content, BASF Company, Ludwigshafen, Germany) were used as surfactants to stabilize the droplets. To initiate the polymerization, azobisisobutyronitrile (AIBN, purity 98%, Sigma Aldrich) was used as an oil soluble thermal initiator. A monofunctional RAFT agent 2-(((dodecylthio)carbonothioyl)thio)propanoic acid (BM1430) was purchased from Boron Molecular and used to mediate the polymerization. All the chemicals were used as received. Deionized MilliQ water was used as polymerization media. To quench the reaction in the samples withdrawn from the reactor at certain time intervals, 1 wt.% hydroquinone (HQ, purity 99%, Fisher Scientific, Madrid, Spain) water solution was used. Tetrahydrofuran (THF, 99.9% GPC, Scharlab, Barcelona, Spain) was used as solvent for the GPC analysis.

### 2.2. Methods 

#### 2.2.1. Synthesis of the First A Block: Batch Miniemulsion Polymerization

RAFT miniemulsion polymerization using an asymmetric RAFT agent was utilized for the synthesis of the polystyrene (pSt) “A” hard block at 30% solids content in water. The following procedure was employed for the miniemulsion preparation. The oil and the water phases were prepared separately and later mixed. First the water phase was prepared by dissolving the surfactant Dowfax 2A (2 wt.% based on styrene monomer, BOM), Disponil A3065 (1 wt.% BOM), and the buffer NaHCO_3_ (0.16 wt.% BOM) in deionized water. The mixture was agitated for 10 min in a beaker using a magnetic bar until homogeneous solution was produced. The costabilizer, stearyl acrylate (SA, 8 wt.% BOM), the RAFT agent (BM1430), and the initiator (AIBN) were dissolved in styrene to form the oil phase. SA is a very hydrophobic monomer, known in the literature as a very effective costabilizer that prevents the Oswald ripening in the miniemulsion [15]. After ensuring a good mixing of both phases, the coarse emulsion was ultrasonicated for 15 min using a Dr. Hielscher GmbH, 400 W (amplitude 70% and 50% duty cycle) sonicator. The sonication was performed under magnetic stirring in an ice-water bath to avoid overheating and possible initiation of the polymerization. The miniemulsion was then transferred to a jacketed batch reactor and purged with nitrogen for 30 min under agitation to eliminate the dissolved oxygen. The temperature was increased to 70 °C and when the desired temperature was reached, time 0 was marked. Samples were taken at different time intervals to measure conversion by gravimetry and molecular weight distribution (MWD) by size exclusion chromatography (SEC) and they were quenched with 1 wt.% hydroquinone (HQ) water solution to stop the polymerization. The reaction was performed for 360 min. Then the temperature was decreased to 25 °C and the final latex (polystyrene A block) was collected and filtered. 

#### 2.2.2. Synthesis of the Second Block: Semi-Batch Emulsion Polymerization 

The synthesized polystyrene “A” block was used as a seed for the synthesis of the “B” block. The “B” block was formed by feeding the monomer (2EHA) as a pre-emulsion for 3 h and polymerization was continued for 2 h batch wise to reach higher conversion. The total solids content of the AB block copolymers synthesized was 30%. The pre-emulsion was made of monomer, water, and the surfactants (Dowfax 2A and Disponil A3065). The total amount of surfactants was kept constant at 3 wt.% based on all monomers. An additional amount of AIBN dissolved in monomer was added once the temperature was increased to 70 °C to start the polymerization. The initiator added was calculated based on the amount of the moles of the RAFT agent present in the seed and was kept constant at a mol ratio of RAFT:initiator = 2:1. To remove the dissolved oxygen, five vacuum (300 mbar) N_2_ (1.5 bar) cycles were applied to the seed and the pre-emulsion prior to polymerization. Moreover, a flow of N_2_ was maintained during polymerizations as well. The initiator dissolved in monomer was purged with N_2_ for 10 min too. Furthermore, nitrogen flow was kept during polymerization as well. At the end of the reaction, the temperature was decreased, and the latex was filtered and collected.

#### 2.2.3. Characterization

Molecular weight distribution and the average molecular weight of the obtained latexes were determined by size exclusion chromatography-gel permeation chromatography (SEC-GPC) at 40 °C with refractive index (RI) and UV detectors. The setting consisted of a pump, an autoinjector (Agilent LC-1260 ALS, Santa Clara, CA, USA) RI detector (Agilent LC- 1260 RID, Santa Clara, CA, USA), a UV detector (Agilent LC- 1260 VWD), and two identical columns in series (Agilent, Polypore 7.5 mm × 300 mm) with molecular weight (MW) range of 200 to 2,000,000 g/mol. The analyses were performed with THF as solvent at a flow rate of 1 mL min^−1^. The polymer was dissolved in THF and filtered (polyamide filter Φ = 0.20 μm). The Mn values obtained were based on calibration with polystyrene standards.

Monomer droplet and particle sizes were obtained using a NANO-flex particle sizer from Microtrac (Haan, Germany) using 780 nm laser light (3 mW) at a 180° scattering angle. Measurements were done with samples diluted to the required concentration with demineralized water at room temperature. Reported average droplets and particle diameters were based on a volume fraction. Three measurements were done, each of them analyzed in 3 runs of 30 s each.

DSC (Differential Scanning Calorimetry) measurements of the latex films dried at room temperature were measured on Q1000, TA Instruments (Hüllhorst, Germany). The scanning cycles consisted of first cooling to −80 °C at 10 °C min^−1^ (isothermal for 2 min), then heating to 150 °C (isothermal for 2 min); second cooling to −80 °C (isothermal for 2 min) at 10 °C min^−1^, then heating to 150 °C at 10 °C min^−1^ and cooling again to 25 °C. The second heating cycle was used for the determination of the Tg.

DMTA (Dynamic Mechanical Thermal Analysis) measurements were performed in tensile geometry on 0.5–0.8 mm thick films using Q800, TA Instruments, at a heating rate of 4 °C min^−1^, frequency of 1 Hz, and at constant strain.

Morphology of the block copolymers films was investigated using transmission electron microscopy (TEM) and atomic force microscopy (AFM). For the TEM investigations of latex particles, block copolymer dispersions were embedded in Natrosol HR 250 = hydroxyethylcellulose (HEC) and stained with RuO_4_. For analysis of the cast films, ultrathin cross-sections (about 100 nm thick) of RuO_4_ stained films, which were dried at different conditions, were prepared with a cryo ultramicrotome (Leica UC 7, Wetzlar, Germany). The sections were examined with a Zeiss Libra 120 microscope (Carl Zeiss, Oberkochen, Germany) with an omega filter operating at an accelerating voltage of 120 kV in elastic mode. 

The morphology of the AB hard-soft block copolymers was further analyzed using Bruker Dimension Icon AFM (Bruker, Karlsruhe, Germany) using Olympus OMCL-AC160TS cantilever (Olympus, Hamburg, Germany) for tapping with a resonant frequency of 300 kHz and spring constant 42 N/m (34–50). Samples were cryocut at −80 °C.

To analyze the adhesive properties, the block copolymer dispersions were drawn to Incada Silk white back folding box board (GC1). Prior to casting, 5 wt.% isopropyl alcohol (based on total dispersion) was added dropwise to the dispersion while mixing to improve wetting and to form continuous and homogenous films as shown in the Supplementary Material (Figure S1). 

The mixture of block copolymer dispersion with 5% isopropyl alcohol was stored overnight to ensure good mixing between components. The paper boards were coated and dried at two different conditions: (i) at room temperature overnight and (ii) at 100 °C overnight. The dry coat weight was approximately 5–6 g/m^2^. After drying overnight, the coated paper boards were heat sealed using a pressure of 100 N/cm for 2 s and 10 s, with various jaw temperature setpoints starting from room temperature, 60 °C, 100 °C, and 150 °C. The coated paper boards were sealed face-to-face to another coated paper board dried at the same conditions (“lacquer to lacquer sealing”). Moreover, the coated paper board was sealed to different uncoated substrates: PVC (polyvinyl chloride), pSt (polystyrene), and PET (polyethylene terephthalate). A heat sealing machine Brugger HSG-C (Brugger, Munich, Germany) was used for sealing the substrates. The machine was equipped with a 20 mm metal top bar to heat the paper board coated with the experimental lacquers and 20 mm lower non-heated silicone bar to press the other substrates to the experimental lacquer. The exact temperature at the sealing point was also monitored. After the surfaces were sealed together and allowed to cool, bond strengths were measured by 180° peel tests performed with a Lloyd LR5K tensile tester (Technex, Wormerveer, The Netherlands), at a peel speed of 150 mm/min. Bond strengths were measured in N/15 mm.

The obtained results were then compared to a benchmark latex available in the market for paper board blister applications, composed of a soft acrylic copolymer (Tg of −30 °C) and a hard alkali soluble resin (Tg of 75 °C).

## 3. Results

### 3.1. RAFT Mediated Miniemulsion Polymerization

The asymmetric trithiocarbonate 2-(((dodecylthio)carbonothioyl)thio)propanoic acid RAFT agent used for the synthesis of the block copolymers is shown in Figure 1. This RAFT agent has an activating Z (–SC_12_H_25_) group based on non-volatile dodecane-thiol and a leaving R (–CH(CH_3_)–COOH) group. Although in small quantities, one could expect that the activating group acts as a surfactant and provides additional stabilization in the miniemulsion process.

**Figure 2:**
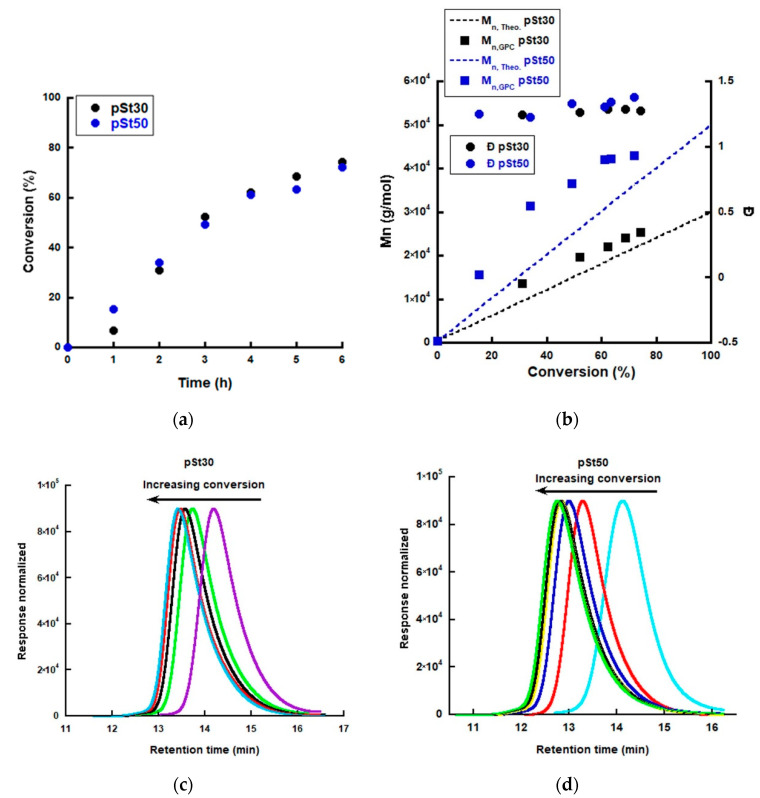
Miniemulsion polymerization of the polystyrene “A” block using RAFT Agent BM1430: (**a**) conversion versus time; (**b**) number average molecular weight and dispersity versus monomer conversion and molecular weight distributions of (**c**) polystyrene (pSt)30 and (**d**) pSt50 obtained by RI refractive index detector in GPC.

Two pSt latexes with different targeted Mn, namely 30,000 and 50,000 g/mol, were synthesized using AIBN as initiator, n(RAFT):n(AIBN) = 4:1 at 70 °C and the results are shown in Figure 2. From the conversion versus time plots (Figure 2a), it can be seen that an almost linear increase was achieved for both homopolymers (pSt30 and pSt50) up to 4 h, above which conversion slowed down. After 6 h of reaction, conversions of 72% and 74% were reached for pSt50 and pSt30, respectively (Figure 2a). Even though a full conversion was not achieved, the reaction was stopped since living blocks were desired, able to be further extended with the second soft monomer. The Mn for both pSt30 and pSt50 increased with conversion. However, it was seen that for both cases the obtained values were higher than the theoretical predictions [24,25]. The effect was more pronounced for the higher targeted Mn (Figure 2b). It should be noted that the amount of costabilizer used in the recipe was not included in the calculation of the targeted Mn. The Mn was higher than the theoretical predictions which suggests that the RAFT agent role was not fully reached [41]. Several proposals have been put forward that suggest ways in which thiocarbonylthio groups are lost or stored during the RAFT process. The fate of intermediate species, their lifetimes, and concentrations as well as rates of fragmentation to R* and Pn* are subjects of intense debate [42,43,44,45,46,47,48]. Nevertheless, regardless of the mechanisms involved, from the data obtained, one could conclude that the number of participating trithiocarbonyl groups is not in accordance to the targeted ones. The molecular weight distribution (Figure 2c,d) of the pSt30 and pSt50 “A” blocks shifted to a higher molecular weight for up to 5 h, after which only a small shift was observed as a result of the enlargement of low Mn chains.

### 3.2. AB Di-Block Copolymer Latex: Hard-Soft Domains

**Figure 3:**
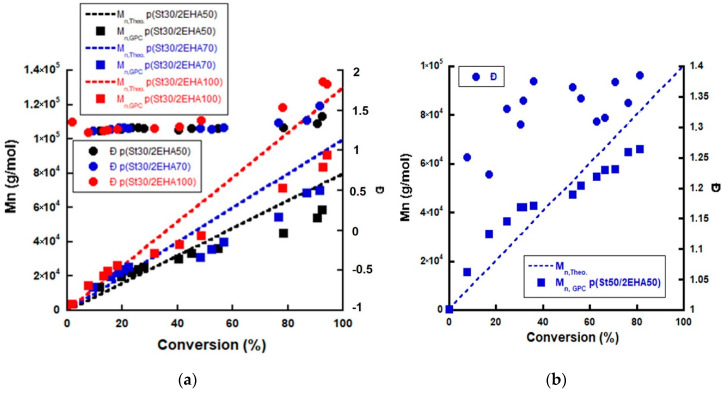
Evolution of the number average molecular weight and dispersity versus monomer conversion for (**a**) p(St30/2EHA50), pSt(30/2EHA70), and p(St30/2EHA100) and (**b**) p(St50/2EHA50) block copolymers.

**Figure 4:**
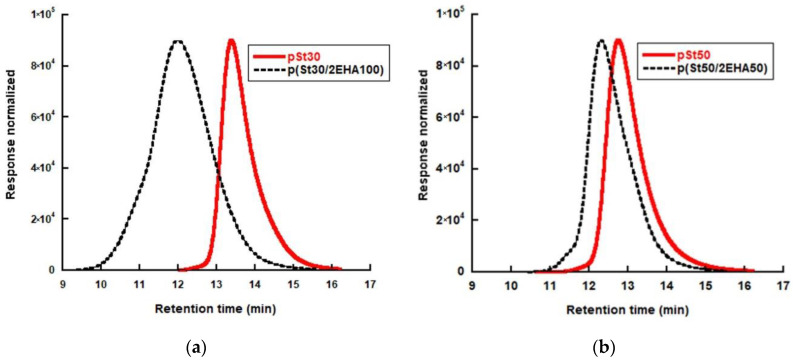
Molecular weight distribution (MWD) of the initial pSt homopolymer and the final block copolymers for (**a**) p(St30/2EHA100) and (**b**) p(St50/2EHA50) as measured by RI detection in GPC.

The pSt30 and pSt50 initial block latexes were extended with 2EHA monomer to form AB block copolymers. The second monomer was fed as a pre-emulsion for 3 h and an additional amount of initiator (AIBN) was added to the system as a shot. The ratio n(RAFT):n(AIBN) was 2:1, calculated based on the moles of RAFT agent present in the seed. Four block copolymers were synthesized with different target molecular weights, named through the text as: p(St50/2EHA50) as an extension of pSt50, and p(St30/2EHA50), p(St30/2EHA70), and p(St30/2EHA100) as extensions of pSt30. The numbers after the letters indicate the targeted molecular weight divided by 1000. The results obtained from the synthesis of the initial blocks and the block copolymers are shown in Table 1. As noticed in Table 1, a second pSt30 latex had to be synthesized to produce p(St30/2EHA100), which presented very similar particle size and just slightly higher Mn compared to the previous pSt30. The second monomer was fed without any removal of the unreacted styrene from the initial block. Considering the reactivity ratios were r1 (styrene) = 1.29 and r2 (2EHA) = 0.73 [49], the soft block was not expected to be pure poly(2EHA), but rather a gradient polymer that still incorporates some styrene units close to the A block and with the end of the chain richer in 2EHA. Nevertheless, the B block is named as p(2EHA) throughout the text for simplicity reasons. From the evolution of Mn versus total monomer conversion (determined by solids content measurements), the block copolymers prepared from pSt30 seed (Figure 3a) showed a linear increase of Mn. Nevertheless, the Mn started to negatively deviate from the theoretical prediction once the second monomer was added. As explained elsewhere [25], this is most likely due to the difference in the Mark–Houwink constants of pSt and 2EHA (the GPC results are based on pSt standards). This effect is less pronounced for p(St50/2EHA50) block copolymer (Figure 4b), since this polymer was richer in pSt compared to the block copolymers starting from a pSt30 seed.

The overall conversion of the synthesized block copolymers was above 90% except for the p(St50/2EHA50), which reached around 81% conversion (Table 1). Moreover, as seen from Table 1, the dispersity (Ð) increased with increasing the targeted Mn, most likely due to branching reactions which are characteristic for acrylates [50,51,52].

The MWDs presented in Figure 4 indicate that the block copolymers have been successfully formed, since not only the average Mn, but all the MWDs for the AB block copolymers shifted to higher molecular weights compared to the initial pSt block. Furthermore, it can be seen that the MWD for p(St30/2EHA100) broadened in the second step, which is in good agreement with the high Ð obtained. 

### 3.3. Thermal Properties of the Initial Homopolymers and Final Block Copolymers 

DSC measurements were performed to determine the glass transition temperatures (Tgs) of the pSt precursor polymers and p(St/2EHA) block copolymers. The Tg values obtained are presented in Table 2, while the thermograms of the final block copolymers are shown in Figure S2 of the Supplementary Materials.

A single glass transition temperature was obtained for both pSt30 and pSt50 initial “A” blocks having different Mn. The Tg values were similar to each other (67.3 °C for pSt30 and 61.0 °C for pSt50), however much lower than the Tg of pure pSt (100 °C). As explained in the experimental part, stearyl acrylate was used as a reactive costabilizer to prevent Oswald ripening in the miniemulsion. The Tg of the polystearyl acrylate is relatively low (−110 °C) [53]. Thus, when this monomer is copolymerized with styrene, we can expect that the Tg will be substantially reduced compared to pure polystyrene, as previously reported [25]. 

The DSC results of the AB block copolymers on the other hand showed two different Tgs. The lower Tg was in the range of −60 °C to −50 °C and corresponded to the soft block composed mainly of p2EHA. The upper Tg was in the range of 70 °C to 80 °C and was associated with Tg of the hard-polystyrene domain. Moreover, from the first derivative of the heat flow versus T curve, we can clearly see that for the block copolymers formed from pSt30 precursor, the area under the peak of the lower Tg was much higher than the area of the higher Tg (Figure S2). On the other hand, the peak areas are almost the same size for the p(St50/2EHA50) block copolymer. This observation corresponds well to the expected microphase separated morphology of the block copolymers with different proportions of soft and hard domains organized in two microphases.

### 3.4. Morphology of the Block Copolymers 

Prior to the analysis of the morphology of the AB block copolymers having hard-soft domains by both TEM and AFM techniques, the theoretical prediction of the morphology obtained from the composition of the block copolymers and the map presented by Bates and Fredrickson (considering full segregation) [54] are presented in Table 3. The composition of the block copolymers was determined by NMR using the method explained by Singha et al. [49] (the NMR spectra of the final block copolymers can be found in Figure S3). Only in the case of p(St50/2EHA50) did the theoretical morphology correspond to the lamellar one; for the rest, the cylinder morphology was expected. 

**Figure 5:**
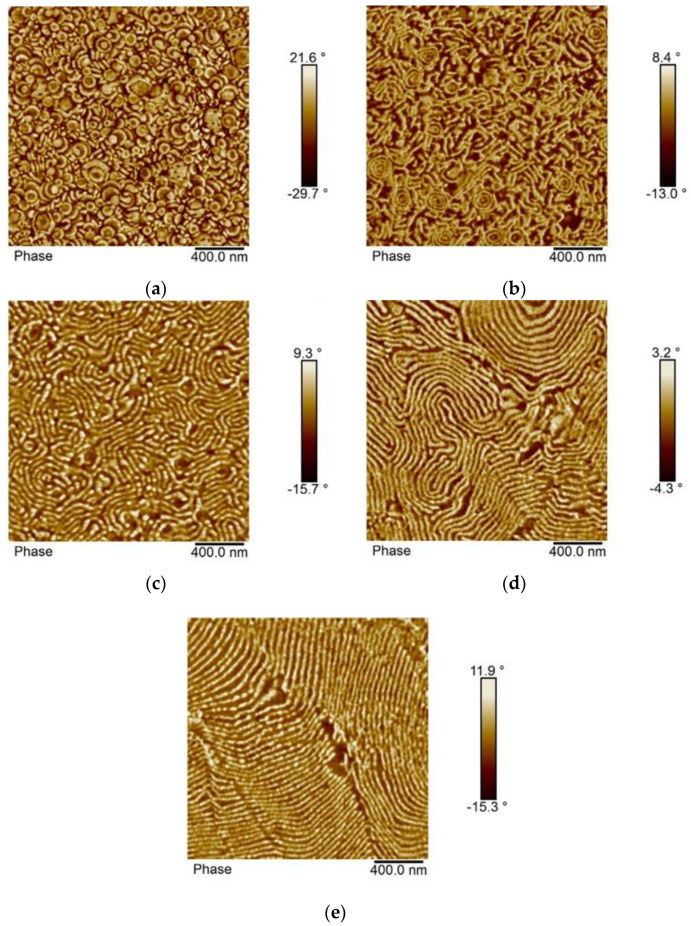
Atomic force microscopy (AFM) phase images of the sample p(St50/2EHA50). (**a**) Top surface of the latex film dried at room temperature; (**b**) cross-section of the latex film dried at room temperature; (**c**) cross-section of the latex film dried at room temperature and annealed at 100 °C for 30 min; (**d**) cross-section of the latex film dried at room temperature and annealed at 100 °C for 96 h; and (**e**) cross-section of a film cast from tetrahydrofuran (THF) solution.

Figure 5 shows the morphology as observed by AFM of samples of p(St50/2EHA50) prepared under different film formation conditions. In the AFM images, the harder pSt domains appear as brighter areas. 

**Figure 6:**
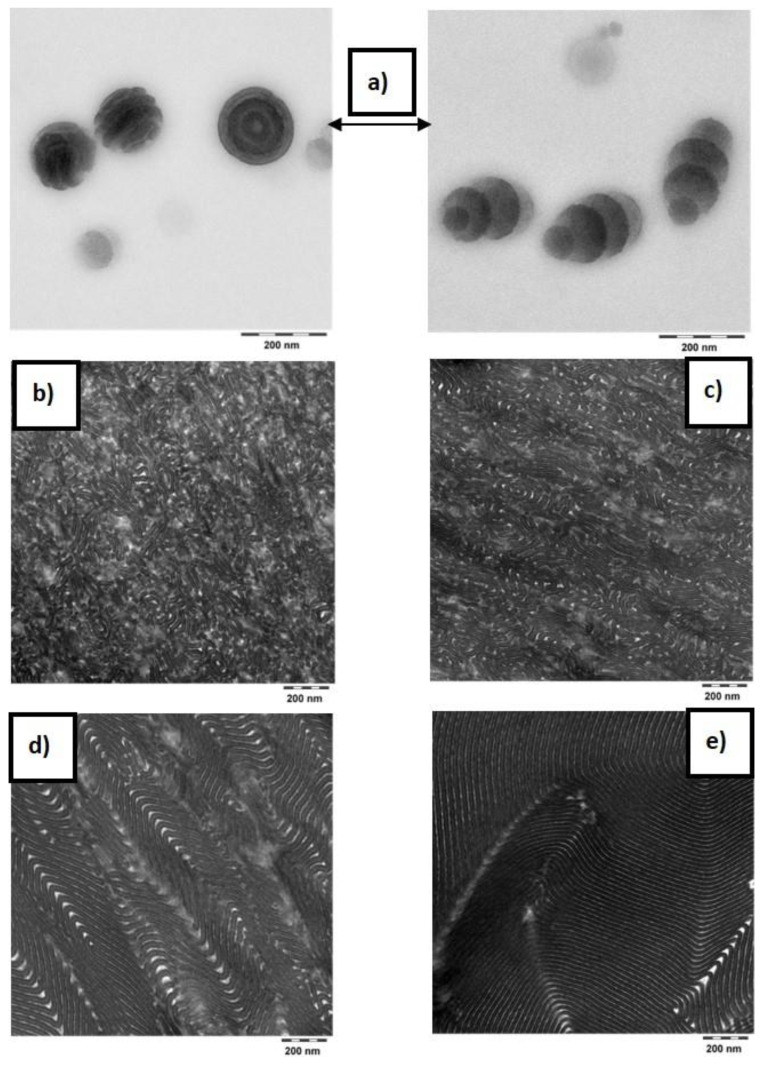
Transmission electron microscopy (TEM) images of the sample p(St50/2EHA50) stained with RuO_4_. (**a**) Particles dispersion embedded in hydroxyethyl cellulose; (**b**) latex film dried at room temperature; (**c**) latex film dried at room temperature and annealed at 100 °C for 30 min; (**d**) latex film dried at room temperature and annealed at 100 °C for 96 h; and (**e**) film cast from THF solution. Scale bar = 200 nm.

The top surface of the latex film dried at room temperature (Figure 5a) shows flattened spheres or cylinders arranged in different directions. The AFM imaging performed on the cross-section of the same film shows the presence of a small portion of spherical particles inside of which an “onion-ring” type lamellar morphology can be distinguished (Figure 5b). In addition, a large portion of short stripes placed in all directions is seen as well. The AFM images were supplemented with TEM images (Figure 6) to verify and complement the interpretation of the morphology analysis obtained by AFM. In the TEM images, pSt domains appear as darker zones. 

The TEM image of the polymer latex dispersion (Figure 6a) clearly shows the presence of two populations of particles: (i) segmented spheres with inner onion-ring morphology and (ii) segmented oblate ellipsoids with polystyrene-rich domains. Self-assembly of block copolymers confined in emulsion droplets, induced by evaporation of solvent from the interior phase of the emulsion, has already been described as a method for the preparation of ellipsoid-shaped and convex lens-shaped particles. This was achieved by tuning the interfacial interactions between the block copolymers particles and the surrounding aqueous solution [55,56,57,58]. One of the strategies to control these interfacial interactions is to use a mixture of surfactants that have selective interactions with each block copolymer domain. When the surfactants at the interface exhibit nonselective or minimal preferential interaction with both blocks, geometry does not affect internal block copolymers nanostructure, but rather the internal structure affects the particle shape to minimize the free energy penalty associated with bending of the block copolymers. In this work, the ellipsoid particles were obtained directly from the synthesis route without use of any solvent. 

To get insight into the kinetics of reordering and equilibration of the microphases, thermal annealing of the films at 100 °C was conducted for two different periods (for 30 min and 96 h). When the annealing was conducted for only 30 min at 100 °C, complete particle coalescence was observed. Moreover, the “onion-ring” structure disappeared, and short range ordered lamellar morphology was obtained Figure 5c and Figure 6c). The pSt lateral lamellae sizes did not exceed more than 400 nm, which suggests that the pSt present only in neighboring particles participated in forming an extended lamella. However, the formation of extended lamellar microphases was more pronounced during prolonged annealing for 96 h (Figure 5d and Figure 6d). At prolonged annealing at 100 °C the pSt lamellae exceeded a range of 1000 nm. Therefore, the ordering of pSt domains occurred over a distance of more than five diameters of the original particles. Dissolving the polymer film first in THF and casting the films from THF solution yielded well-ordered extended lamellar morphology, visible both by AFM (Figure 5e) and TEM (Figure 6e). Based on the NMR results, these block copolymers have around 50 wt.% of hard domains, which is well in line with the theoretical predicted lamellar morphology according to the diagram for asymmetric AB type block copolymers. 

**Figure 7:**
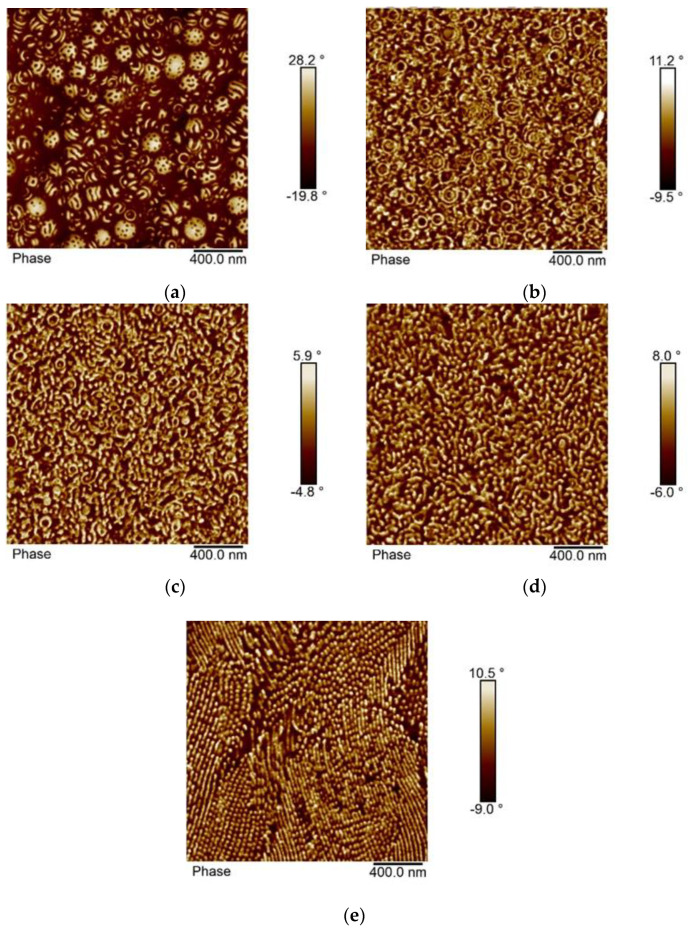
AFM phase images of the sample p(St30/2EHA50). (**a**) Top surface of the latex film dried at room temperature; (**b**) cross-section of the latex film dried at room temperature; (**c**) cross-section of the latex film dried at room temperature and annealed at 100 °C for 30 min; (**d**) cross-section of the latex film dried at room temperature and annealed at 100 °C for 96 h; and (**e**) cross-section of a film cast from THF solution.

**Figure 8:**
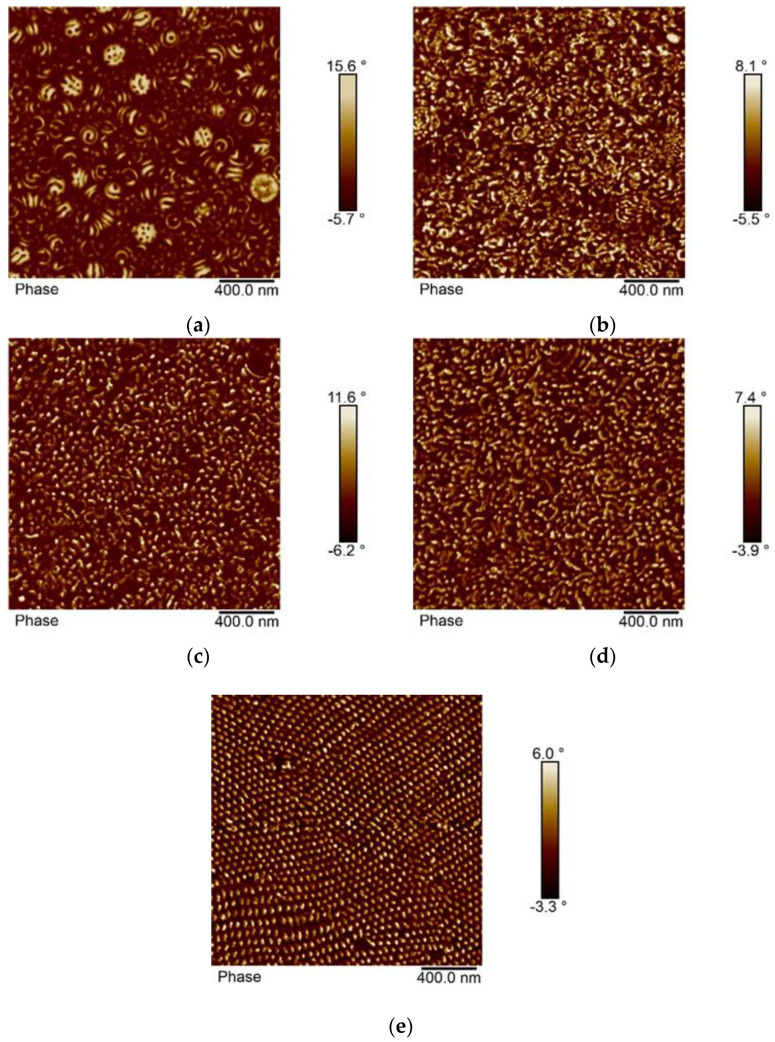
AFM phase images of the sample p(St30-2EHA70). (**a**) Top surface of the latex film dried at room temperature; (**b**) cross-section of the latex film dried at room temperature; (**c**) cross-section of the latex film dried at room temperature and annealed at 100 °C for 30 min; (**d**) cross-section of the latex film dried at room temperature and annealed at 100 °C for 96 h; and (**e**) cross-section of a film cast from THF solution.

**Figure 9:**
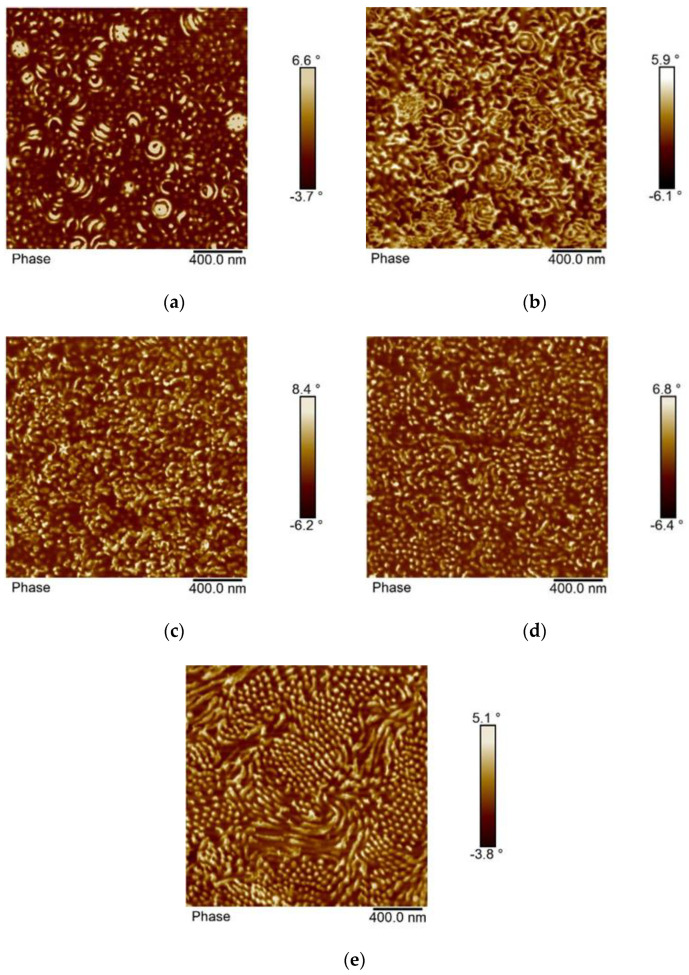
AFM phase images of the sample p(St30/2EHA100). (**a**) Top surface of the latex film dried at room temperature; (**b**) cross-section of the latex film dried at room temperature; (**c**) cross-section of the latex film dried at room temperature and annealed at 100 °C for 30 min; (**d**) cross-section of the latex film dried at room temperature and annealed at 100 °C for 96 h; and (**e**) cross-section of a film cast from THF solution.

The morphology of the block copolymer samples with composition p(St30/2EHA50), p(St30/2EHA70), and p(St30/2EHA100) is shown in Figure 7, Figure 8 and Figure 9. The weight fractions of the hard-polystyrene domains based on the NMR results are 21.1%, 27.6%, and 34.7% for p(St30/2EHA100), p(St30/2EHA70), and p(St30/2EHA50) respectively. The measured weight fractions of pSt can be taken as a good approximation for the volume fractions. Thus, according to the AB block copolymers morphology diagram, we should expect a bulk morphology with cylinder-type microphases of PSt. The AFM imaging of the top surface of the latex films dried at room temperature (Figure 7a, Figure 8a, and Figure 9a) shows the presence of spherical microphase separated particles whose surfaces are composed of both polymer phases. The pSt hard domains three-dimensionally twist around the soft p(2-EHA) phases in a helical axis. The higher the pSt content in the sample, the more pronounced the helix. 

The cross-sections of the polymer films dried at room temperature (Figure 7b, Figure 8b, and Figure 9b) show that most of the particles have an inner structure with one or two layers of pSt and only a few particles show onion-ring morphology. Moreover, polystyrene domains spread all over the matrix as white spots are visible. Thermal annealing of the polymer films led to rearrangement of the hard domains. The effect was especially pronounced for a longer annealing period and for the block copolymers with a higher proportion of soft domains (Figure 8d and Figure 9d.). Dissolving the polymer film in THF completely erased the thermal history of the sample, and in the films cast from THF solution, a clear phase separation was observed where cylinders were placed in either horizontal or perpendicular direction parallel to each other (Figure 7e, Figure 8e, and Figure 9e.).

### 3.5. Viscoelastic Properties of AB Hard-Soft Block Copolymers

**Figure 10:**
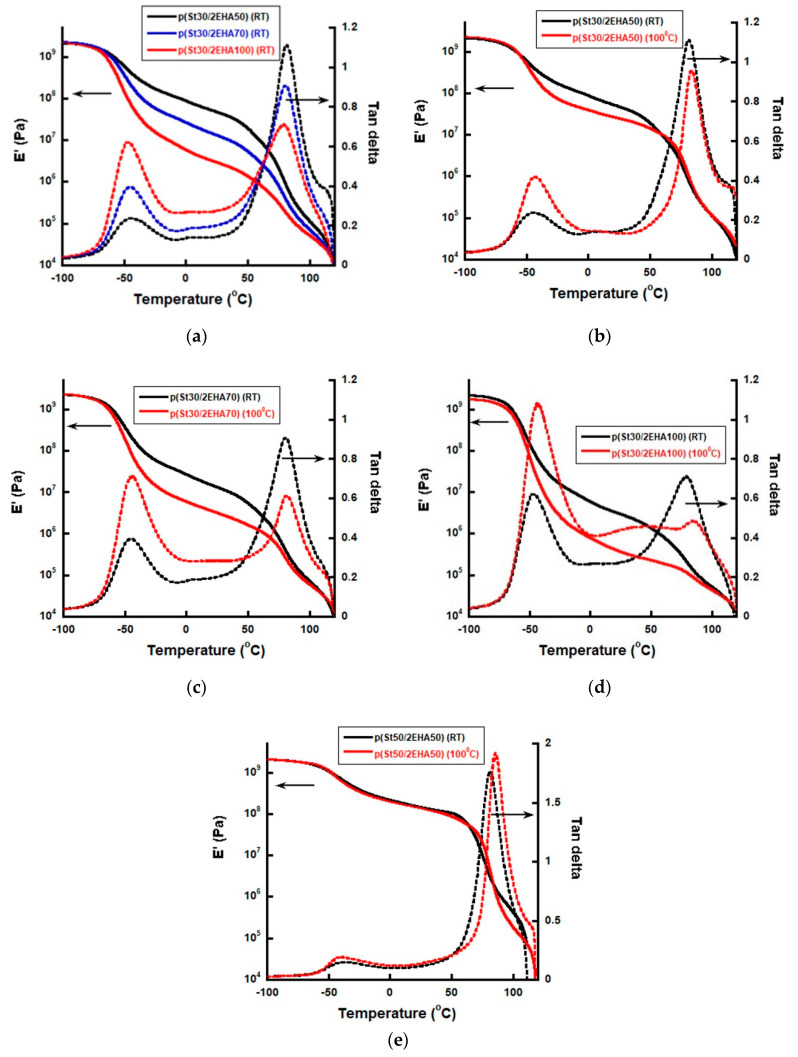
Viscoelastic properties of the block copolymers: (**a**) p(St30/2EHA50), p(St30/2EHA70) and p(St30/2EHA100) dried at RT; (**b**) p(St30/2EHA50) dried at RT and 100 °C; (**c**) p(St30/2EHA70) dried at RT and 100 °C; (**d**) p(St30/2EHA100) dried at RT and 100 °C; (**e**) p(St50/2EHA50) dried at RT and 100 °C. Solid lines = dependence of elastic modulus on temperature; dashed lines = dependence of tan δ on temperature.

The mechanical properties of films of the AB hard-soft block copolymers were investigated by means of DMTA measurements and the results are presented in Figure 10a–e. The solid line shows the temperature dependent variation of the storage modulus and the dashed line shows the behavior of tan δ. Two different transition temperatures, one at a lower temperature corresponding to the Tg of the soft rubbery block and one at a higher temperature corresponding to the Tg of the hard domains, could be distinguished. Moreover, a rubbery plateau in-between them was visible. 

Figure 10a presents the viscoelastic properties of the films cast at room temperature. It is seen from the figure that the elastic modulus decreases, and the damping properties increase as the length (Mn) of the soft p2EHA block in the AB block copolymers increases. Moreover, the elastic modulus decreases substantially in the rubbery plateau in the temperature range between −30 °C and 30 °C.

The viscoelastic properties of the polymer films dried at room temperature were compared to the ones annealed at higher temperature (Figure 10b–e). As a general trend, we observed a visible decrease of the elastic module in the rubber plateau region of the films annealed at 100 °C, compared to the corresponding films dried at room temperature. This drop of the elastic modulus in the rubber plateau upon annealing is more pronounced for the block copolymers with lowest % wt. of pSt. The effect is almost negligible for the sample with the highest amount of pSt domains of ~50 wt.% (Figure 10e). Different to the other block copolymers, this sample p(St50/2EHA50) displayed extended pure lamellar micromorphology after annealing, while for the rest of the block copolymers, pSt domains isolated in a p2EHA soft matrix could be observed.

### 3.6. Heat Sealing Properties of the Block Copolymers

**Figure 11:**
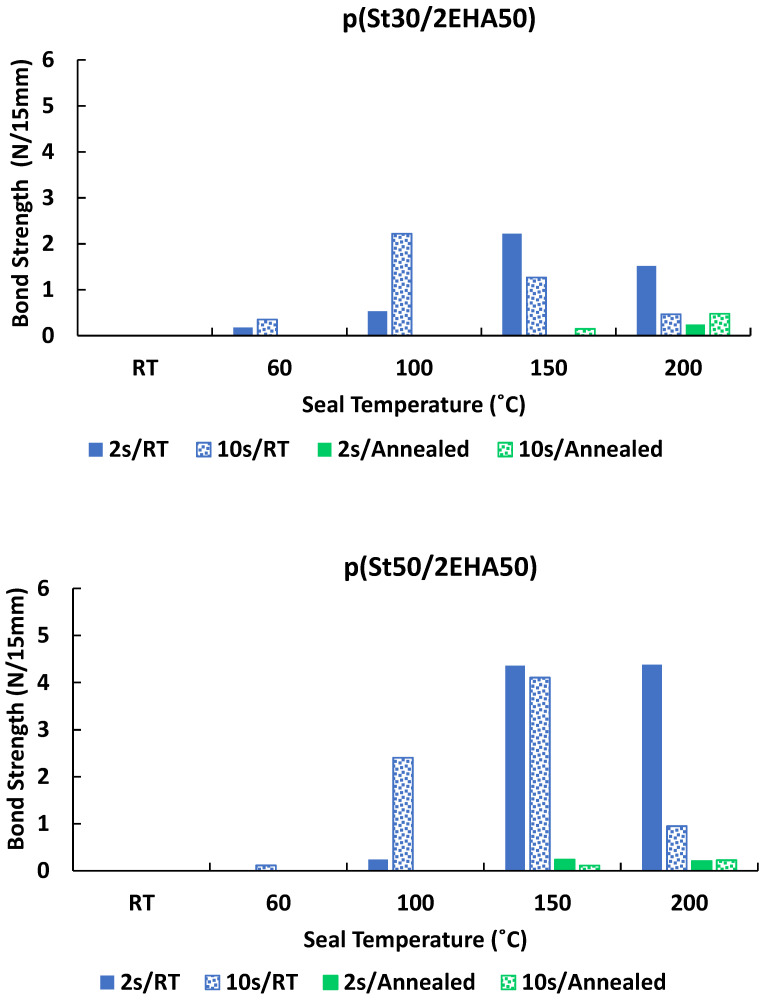
Bond strength of hard-soft AB block copolymer dispersions coated on paper (paper-to-paper seal). Sealing experiments done at dwell times of 2 s and 10 s. Coating preparations: RT = coated papers where dried at room temperature; annealed = coated papers where in addition stored at 100 °C overnight.

As explained in the introduction, the heat seal capacity of the synthesized waterborne block copolymers was analyzed as a possible application area for these thermoresponsive block copolymers. The heat-sealing properties of the p(St30/2EHA50), p(St30/2EHA70), p(St30/2EHA100), and p(St50/2EHA50) block copolymers with different Mn were investigated in paper-to-paper applications initially (lacquer-to-lacquer sealing). At first sight, we noticed that the samples with higher content of soft domains—p(St30/2EHA70) and p(St30/2EHA100)—showed a visible tackiness and certain bond strengths already at room temperature. This behavior makes them unsuitable for usage in heat sealable lacquers, as this will lead to difficulties in unwinding the coated substrates and generate surface defects during the converting process. Therefore, we report here only the results of the bond strengths of the paper-to-paper heat sealable lacquers for p(St30/2EHA50) and p(St50/2EHA50) (see Figure 11). The blue bars represent the peel force or bond strength obtained from the drawdowns dried at room temperature and the green bars represent the peel force obtained from the drawdowns annealed at 100 °C overnight. On the other hand, the solid filled bars are the data obtained from the substrates pressed for 2 s and the patterned bars represent the data obtained from the films pressed for 10 s. 

The results presented in Figure 11 show that the highest pSt content samples—p(St50/2EHA50) and p(St30/2EHA50)—with 48.8 wt.% and 34.7 wt.% of pSt, respectively (obtained from the NMR results), show no tackiness at room temperatures. Furthermore, it can be observed that at sealing temperatures of 60 °C and 100 °C, higher bond strengths are obtained for higher dwell times for the non-annealed lacquers. When the top bar was heated to 60 °C and 100 °C at 2 s dwell time, the real temperature in the seal appeared only at 40 °C and 65 °C, respectively, as measured with a thermocouple (see Figure S4). Thus, the temperature is not high enough to soften the hard domains and to allow enough interdiffusion so that both coating layers can effectively mix and create a solid bonding after cooling. On the other hand, if the sealing temperature is 150 °C, higher bond strengths are obtained, both for dwell times of 2 and 10 s. An interesting phenomenon observed at temperatures above 100 °C, is that the samples sealed at higher dwell times (10 s) showed lower bond strength than the ones sealed at lower dwell times (2 s). Most likely these block copolymers show lower melt viscosity at higher temperatures, and thus when pressed, the polymer film diffuses into the pores of the paper. Thus, there is lower effective thickness and less surface contact. 

In addition, it was surprising to see that the previously annealed samples showed no or much lower bond strengths. This is most likely because of two ongoing effects during annealing: (i) migration of surfactant to the surface and/or (ii) substantial phase separation and rearranging of the domains during annealing, such that higher temperatures and/or more time is needed to achieve adhesiveness. As inferred from Figure 5, Figure 6 and Figure 7, going from the non-equilibrium morphology of the film obtained after film formation at room temperature to a more equilibrium one seems easier in the heat seal experiment, than moving from a morphology that probably is in equilibrium after being annealed at 100 °C overnight. 

Moreover, the use of samples p(St30/2EHA50) and p(St50/2EHA50) as paper-to-paper heat seal lacquers was compared to the benchmark latex using a dwell time of 2 s at 150 °C. It was seen that even if sample p(St30/2EHA50) (2.22 N/15 mm) showed lower bond strength compared to the benchmark (5.13 N/15 mm), sample p(St50/2EHA50) (4.35 N/15 mm) showed a performance approaching the benchmark. 

**Figure 12:**
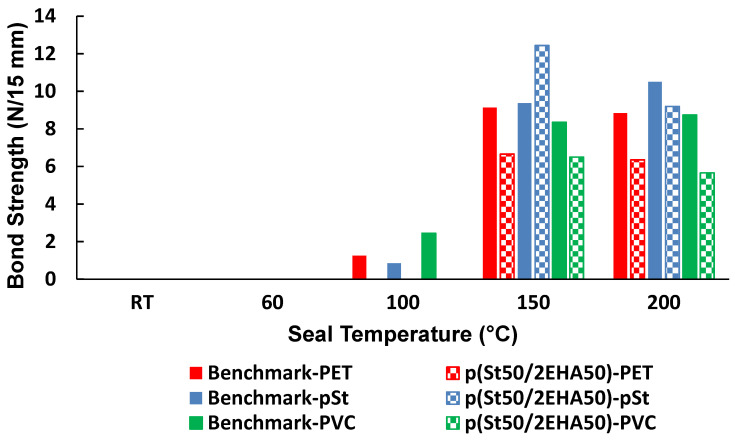
Bond strength of a hard-soft AB block copolymer compared to the benchmark dispersion. Testing of coated papers in sealing to non-coated plastic films: PET (red), pSt (blue), PVC (green) substrates.

In order to further analyze the heat seal capabilities of p(St50/2EHA50) dispersion, it was coated on a paper board and sealed for 2 s to three different substrates: PET, pSt, and PVC; the results are shown in Figure 12. 

We can see that the block copolymers did not seal onto the substrates at 100 °C and the benchmark latex showed only very low bond strength manifested as pealing failure at this temperature. This could be related to the low molecular weight alkali soluble resin in the composition of the benchmark latex, which activates at lower temperature and thus manifests a certain bond strength already at lower temperature. Furthermore, the block copolymer dispersion showed lower bond strength compared to the benchmark latex for PET and PVC substrates at 150 °C. On the other hand, higher bond strength values were obtained compared to the benchmark latex when paper cardboard was sealed to pSt substrates at 150 °C. This is most likely due to the higher compatibility of the block copolymer with pSt rather than with PET and PVC.

## 4. Conclusions

Two-step reversible addition fragmentation chain transfer polymerization and an asymmetric 2-(((dodecylthio)carbonothioyl)thio)propanoic BM1430 RAFT agent were used to synthesize a series of waterborne AB block copolymers containing hard and soft domains. The synthesis of the hard-polystyrene domains was done first using miniemulsion polymerization and then 2EHA soft blocks were incorporated by emulsion polymerization. Successful formation of the AB block copolymers was proven by MWD shift to higher Mn and a linear increase of Mn versus conversion. The block copolymers on the other hand showed two Tgs corresponding to the two microdomains present in the system. 

When the waterborne block copolymer latexes were dried at room temperature, the particle deformation and coalescence were not complete, and the nano-phase separation originating from the particle morphologies was partly preserved and could still be detected in the films, as seen by TEM and AFM. However, it was seen that 30 min of annealing at 100 °C already erased the particle morphology, but the newly formed microphase ordering typically only ranged over a length scale of about two or three particle diameters. Annealing at prolonged times at 100 °C led to more ordered microphase separated morphologies, which appeared equal to the equilibrium morphologies obtained by casting the films from a good solvent. 

Furthermore, it was shown that the viscoelastic properties of the block copolymers are greatly influenced by the block copolymer composition and the annealing treatment. In general, thermal annealing reduced the elastic modulus in the plateau region and this effect was more prominent for the block copolymers with larger soft segments. Adhesive properties in the form of paper-to-paper heat seal lacquers were evaluated, and it was shown that the dwelling time and composition of the block copolymers greatly influenced the values obtained. The highest bond strength for paper-to-paper heat seal lacquers was obtained for the p(St50/2EHA50) block copolymer. The achieved bond strength values could not reach the performance of the commercial benchmark latex. However, when the coated paper was sealed to pSt substrate, the bond strength values exceeded that of the benchmark latex. The example of the application in heat seal lacquers demonstrates that the thermoresponsive properties of such waterborne block copolymer systems can be tuned by changing the relative amount of hard and soft blocks to achieve or even encompass the performance of relevant commercial benchmark polymers. 

## Figures and Tables

**Table 1:** Conversion, average particle size (dd: diameter of the droplets; dp: diameter of the particles), Mn, and Ð at the end of reaction for pSt homopolymers and p(St/2-ethylhexyl acrylate (2EHA)) block copolymers.

Reactions	Conversion (%)	M_n,GPC_ (g/mol)	Ð	dd (nm)	dp (nm)	Conversion (%)	dp (nm)	M_n,GPC_ (g/mol)	Ð
	pSt Homopolymers					P(St/2EHA) Block Copolymers			
p(St50/2EHA50)	72.0	36,121	1.37	98.6	115.3	81.2	150.4	65,980	1.38
p(St30/2EHA50)	74.2	22,364	1.27	100.6	121.1	92.5	160.6	58,611	1.42
p(St30/2EHA70)	74.2	22,364	1.27	100.6	121.1	91.4	165.0	70,103	1.55
p(St30/2EHA100)	78.1	23,509	1.27	89.9	126.2	94.2	171.3	90,834	1.83

**Table 2:** Thermal properties of the pSt initial blocks and p(St/EHA) block copolymers obtained by DSC measurements.

Material Code	Tg_1_ (°C)	Tg_2_ (°C)
DSC analysis of the samples dried at 23 °C		
pSt30	-	67.3
pSt50	-	61.0
p(St30/2EHA50)	−60.5	79.6
p(St30/2EHA70)	−60.5	78.3
p(St30/2EHA100)	−61.6	79.5
p(St50/2EHA50)	−50.4	69.9

**Table 3:** Block copolymer composition determined by ^1^H NMR spectroscopy and theoretical morphology predicted.

Material Code	Mole Fraction		Weight Fraction		Theoretical Morphology
	% St	%2EHA + SA	%St + SA	%2EHA	
p(St50/2EHA50)	63.5	36.5	48.8	51.2	L
p(St30/2EHA50)	48.9	51.1	34.7	65.3	C
p(St30/2EHA70)	40.4	59.6	27.6	72.4	C
p(St30/2EHA100)	32.4	67.6	21.1	78.9	C

L = lamellar; C = cylinder; SA = stearyl acrylate.

## Supplementary Materials

The following are available online at https://www.mdpi.com/2073-4360/12/6/1253/s1, Figure S1: Block copolymer dispersion cast onto aluminum foils with bar coater with and without 5 wt.% isopropyl alcohol wetting agent, Figure S2: DSC thermograms of the p(St/2EHA) block copolymers, Figure S3: NMR spectra of the final block copolymers, Figure S4: In-seal temperature profile measured for a commercial standard reference polymer dispersion.
