# Supplementary material for: Design of Waterborne Asymmetric Block Copolymers as Thermoresponsive Materials
Source: Polymers (Basel). 2020 May 30;12(6):1253. doi: 10.3390/polym12061253 (PMC7361867; doi:10.3390/polym12061253)
Supplement: Supplementary file 1 [file polymers-12-01253-s001.pdf]

Supplementary Material

Design of Waterborne Asymmetric Block Copolymers as Thermoresponsive Materials

Gordana Siljanovska Petreska [1,2], Christof van Sluijs [3], Clemens Auschra [2] and Maria Paulis [1,*]

[1] POLYMAT, University of the Basque Country UPV/EHU, Joxe Mari Korta Center, Avda. Tolosa 72, 20018 Donostia-San Sebastián, Spain; gordana.siljanovska-petreska@basf.com
[2] BASF SE, 67056 Ludwigshafen, Germany; clemens.auschra@basf.com
[3] BASF Nederland BV, 8440 AJ Heerenveen, The Netherlands; christof.van-sluijs@basf.com
* Correspondence: maria.paulis@ehu.eus

To improve the wetting of the block copolymer dispersions and to form continuous and homogenous films, prior to casting, 5 wt % isopropyl alcohol (based on total dispersion) was added dropwise to the dispersion while mixing. The drawdowns shown in Figure S1 were obtained on aluminum foil for a better contrast. The addition of the alcohol improved efficiently the wetting and homogeneity of the films.

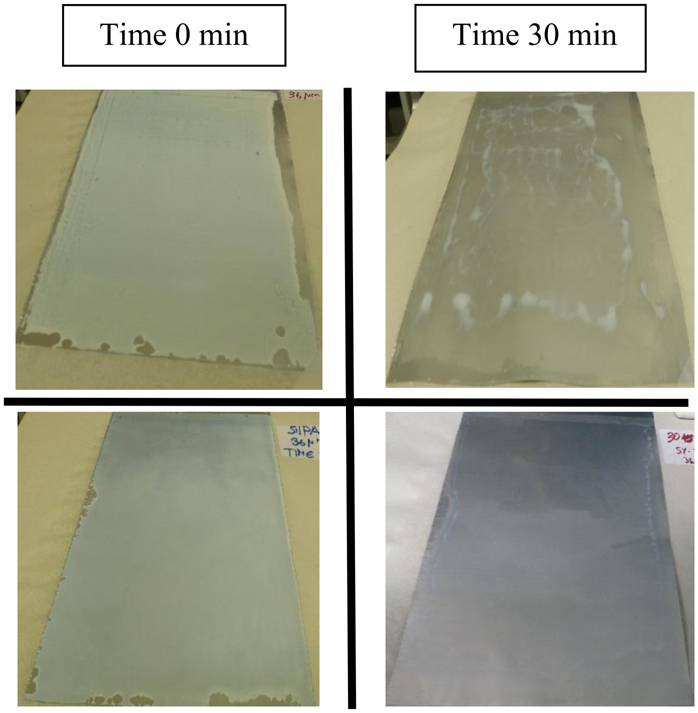

Figure S1. Emulsion polymers cast onto aluminum foils with bar coater (defined film with a wet layer thickness of 36 µm): AB block copolymers dispersion (upper images) and AB block copolymers dispersion + 5 wt% isopropyl alcohol (lower images)

The DSC curves (Heat Flow and Derivative of the Heat Flow) of the final block copolymers are presented in Figure S2. 1H NMR spectra of the final block copolymers are presented in Figure S3.

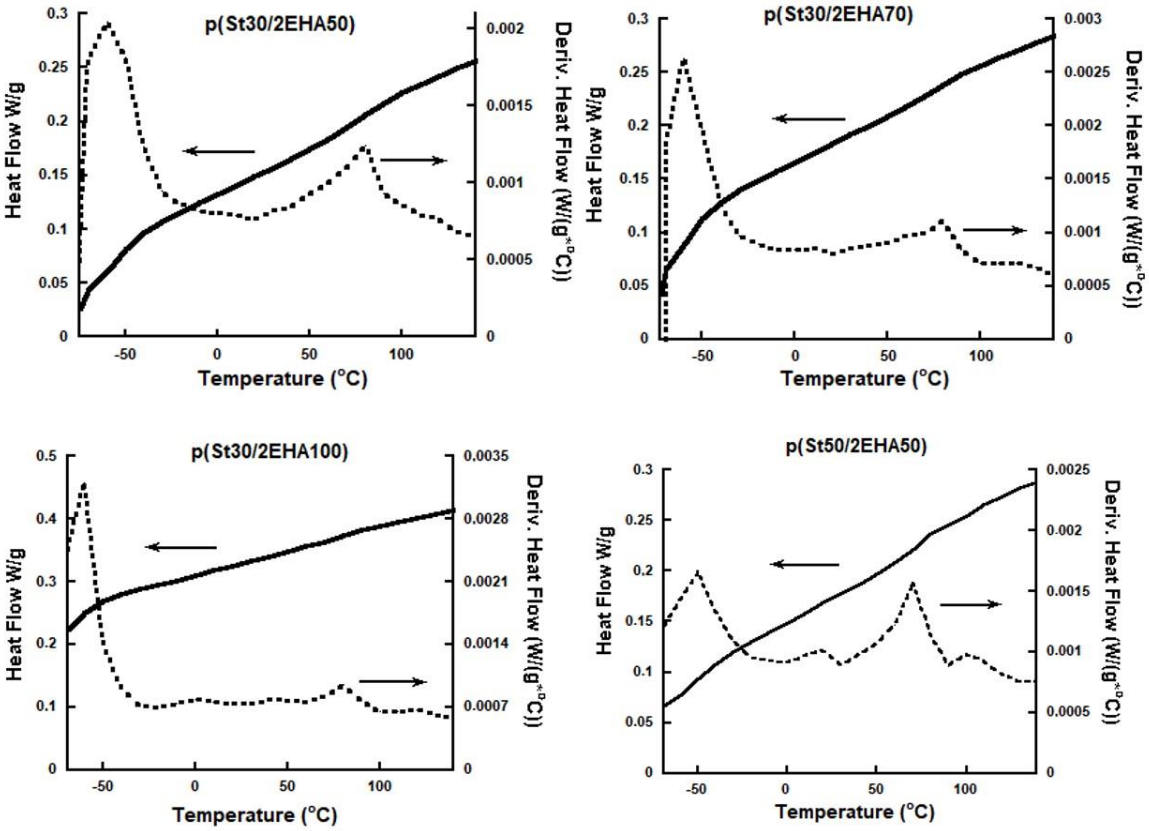

Figure 2. DSC thermograms of the p(St/2EHA) block copolymers measured at a heating rate of 10 °C/min.

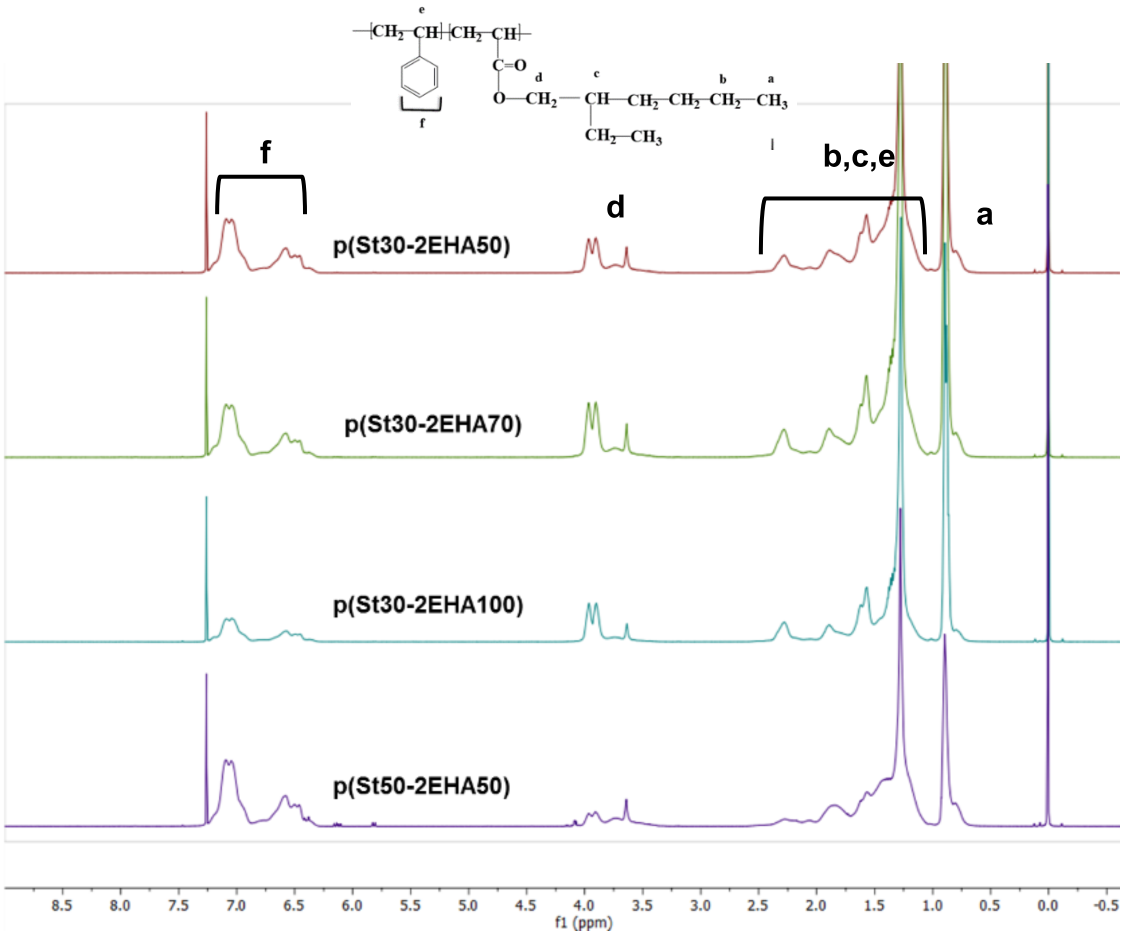

Figure S3. ^{1}H NMR spectra of the p(St/2EHA) block copolymers.

The temperature in the seal area was measured during sealing and a temperature profile over time was obtained using a coated paper board with a commercial standard polymer dispersion sealed on PET substrate, using two different dwell times (2 s and 10 s), by which the coated paper sheet and the non-coated PET sheet are pressed together. The results are shown in S1. It can be seen from the figure that the in-seal temperature differs from the target set temperature of the machine, and this difference is higher for lower dwell times. An increase from 20 ˚C to 40 ˚C with dwell time increase from 2 s to 10 s was observed for different target temperatures. For example, even though the display temperature of the machine for the sealing tool was 200 ˚C and the substrates were pressed for 10 s, the actual temperature that the substrate reached was only 160 ˚C (see upper black dotted curve in Figure S4). This is because certain time is needed to transfer the heat from the hot plates of the machine through the cardboard to the lacquer layer.

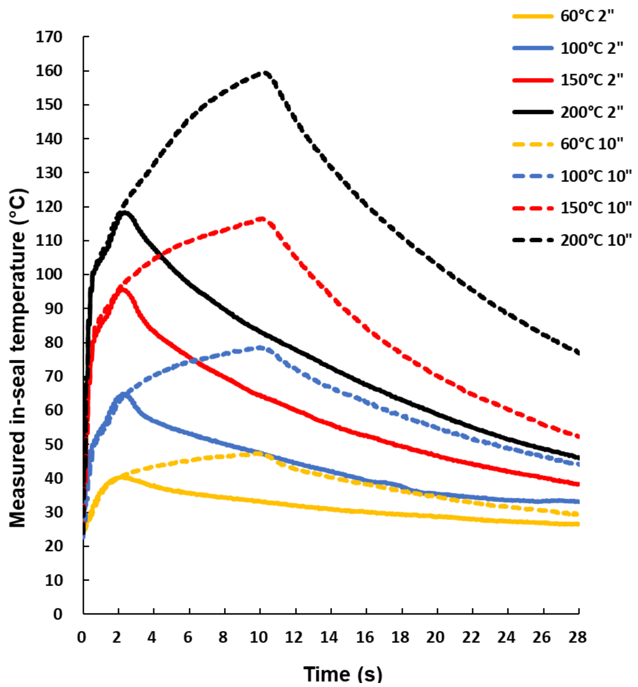

Figure S4. In-Seal temperature profile measured for a commercial standard reference polymer dispersion; variations of real seal temperatures versus time for different target set temperatures and two different dwell times (2 s and 10 s) by which the coated paper sheet was pressed against non-coated PET.
